# Kinetic Changes of Peripheral Blood Monocyte Subsets and Expression of Co-Stimulatory Molecules during Acute Dengue Virus Infection

**DOI:** 10.3390/pathogens10111458

**Published:** 2021-11-10

**Authors:** Sakaorat Lertjuthaporn, Rassamon Keawvichit, Korakot Polsrila, Kasama Sukapirom, Ampaiwan Chuansumrit, Kulkanya Chokephaibulkit, Aftab A. Ansari, Ladawan Khowawisetsut, Kovit Pattanapanyasat

**Affiliations:** 1 Center for Research and Innovation, Faculty of Medical Technology, Mahidol University, Nakhon Pathom 73170, Thailand; sakaorat.ler@mahidol.edu; 2 Department of Clinical Pathology, Faculty of Medicine Vajira Hospital, Navamindradhiraj University, Bangkok 10300, Thailand; rassamon@nmu.ac.th; 3 Research Department, Faculty of Medicine Siriraj Hospital, Mahidol University, Bangkok 10700, Thailand; kokchou@gmail.com; 4 Center of Excellence for Microparticle and Exosome in Diseases, Research Department, Faculty of Medicine Siriraj Hospital, Mahidol University, Bangkok 10700, Thailand; kasama.suk@mahidol.ac.th; 5 Department of Pediatrics, Faculty of Medicine Ramathibodi Hospital, Mahidol University, Bangkok 10400, Thailand; ampaiwan.jua@mahidol.ac.th; 6 Department of Pediatrics, Faculty of Medicine Siriraj Hospital, Mahidol University, Bangkok 10700, Thailand; kulkanya.cho@mahidol.ac.th; 7 Department of Pathology and Laboratory Medicine, Emory University School of Medicine, Atlanta, GA 30322, USA; pathaaa@emory.edu; 8 Department of Parasitology, Faculty of Medicine Siriraj Hospital, Mahidol University, Bangkok 10700, Thailand

**Keywords:** dengue infection, monocyte, flow cytometry, co-stimulation

## Abstract

Monocytes, one of the main target cells for dengue virus (DENV) infection, contribute to the resolution of viremia and to pathogenesis. We performed a longitudinal study by a detailed phenotypic comparison of classical (CD14++CD16−, non-classical (CD14+CD16++) and intermediate (CD14++CD16+) monocyte subsets in blood samples from dengue fever (DF) to the severe dengue hemorrhagic fever (DHF) and healthy individuals. Various costimulatory molecules of CD40, CD80, CD86 and inducible costimulatory ligand (ICOSL) expressed on these three monocyte subsets were also analyzed. DENV-infected patients showed an increase in the frequency of intermediate monocytes and a decrease in the classical monocytes when compared to healthy individuals. Although these differences did not correlate with disease severity, changes during the early phase of infection gradually returned to normal in the defervescence phase. Moreover, decreased frequency of classical monocytes was associated with a significant up-regulation of co-stimulatory molecules CD40, CD86 and ICOSL. Kinetics of these co-stimulatory molecule-expressing classical monocytes showed different patterns throughout the sampling times of acute DENV infection. Different distribution of monocyte subsets and their co-stimulatory molecules in the peripheral blood during acute infection might exacerbate immune responses like cytokine storms and ADE, and future studies on intracellular molecular pathways utilized by these monocyte linages are warranted.

## 1. Introduction

Dengue virus (DENV) infection is one of the most significant emerging diseases that affects nearly 100 million people annually in over 100 countries, occurring predominantly in countries within the tropical and sub-tropical regions [1]. There are four serotypes of DENV (DENV1 to DENV4), and each can cause disease ranging from self-limiting, mild febrile illness of dengue fever (DF) to life-threatening complications of dengue hemorrhagic fever (DHF) and the fatal dengue shock syndrome (DSS) [2,3]. There is a continuum of symptoms from DF to DHF/DSS differentiated physiologically by the degree of vascular permeability and hemostatic disorders [4]. There have been several proposed hypotheses forwarded that attempt to describe the basis for such diverse outcomes of DENV infections. These include exacerbated immune response with varying degrees of elevated levels of cytokines or cytokine storms, as well as antibody-dependent enhancement (ADE) [5,6]. The latter occurs predominantly either in patients with secondary heterologous DENV infections or in infants with primary infections born to mothers with established immunity [7,8,9]. Such findings implicate a role for pre-existing antibodies induced by the primary infection that have waned to non-neutralizing levels. These antibodies upon secondary heterotypic infection potentially lead to viral internalization via Fc receptors and enhanced replication within phagocytic cells followed by massive release of soluble factors capable of affecting endothelial cell damage and loss of platelet integrity that lead to DHF/DSS.

Monocytes are among the major subsets of white blood cell with bean-shaped nuclei and a finely granular cytoplasm containing lysosomes and phagocytic vacuoles. They are the most abundant blood mononuclear phagocytes, and originate from myeloid precursors from the bone marrow and differentiate into myeloid lineage dendritic cells and macrophages in tissue. There are at least three subsets of human blood monocytes that are distinguished by their expression of the lipopolysaccharide membrane receptor (CD14) and the low-affinity Fcγ receptor (CD16) along with intrinsic differences in function [10]. The three subsets include the classical (CD14++CD16−), non-classical (CD14+CD16++) and intermediate (CD14++CD16+) subsets. The classical monocytes produce interleukin-10 (IL-10), IL-8, monocyte chemotactic protein-1 (MCP-1; CCL2) and RANTES (CCL5) upon stimulation with lipopolysaccharide [11,12]. The non-classical monocytes are the predominant subset that synthesize inflammatory mediators such as IL-1β, TNF-α, and CXCL10 or IFN-γ inducible protein-10 in response to DENV infection. They can also detect nucleic acids and viruses via Toll-like receptor 7 (TLR7) and TLR8. Lastly, the intermediate monocytes, unlike the non-classical monocytes, sense ligands expressed by TLR2, TLR4 and TLR8 and possess the potential to synthesize IL-6, IL-8, as well as CCL2, CXCL10, IL-1β, and TNF-α [11,12]. Moreover, with reference to virus-specific immune responses, intermediate monocytes express relatively higher levels of major histocompatibility complex (MHC) class II molecules compared to classical and non-classical monocytes [12,13].

Despite the difficulty of infecting monocytes/macrophages with DENV in vitro without the presence of DENV antibody [14], several studies in both humans and nonhuman primates have indicated that cells of the mononuclear phagocyte lineage are one of the major targets of DENV [14,15,16,17]. This view is supported by the finding of DENV antigen within the Kupffer cells of the liver and alveolar macrophages in biopsies obtained pre- and post-mortem from DHF/DSS patients [18]. However, it is important to note that instead of direct infection, these results could be secondary to phagocytosis of DENV by these cells [17,18]. In our previous study of the potential role of monocytes/macrophages in DENV infection, we examined the phenotypic changes of monocytes in the blood of humans and in the non-human primate model of DENV infection. Results of this cross-sectional study showed that DENV infection leads to an increase in the number of CD14++CD16+ blood monocytes during the early host response to infection [19]. An increase of these intermediate monocytes is followed by a robust expansion of plasmablasts arising from resting B cells accompanied by the synthesis and secretion of immunoglobulin G (IgG) and IgM. These findings thus highlight a role of intermediate monocytes in mediating dengue virus specific adaptive immune response. Given the above information and with the purpose to enhance a greater understanding of the role that the intermediate monocytes play in DENV infection, this previous cross-sectional study was extended to include a longitudinal study in attempts to identify when changes, if any, occur. The longitudinal study included analysis of changes in the frequencies of all three monocyte subsets, classical (CD14++CD16-), non-classical (CD14+CD16++) and intermediate (CD14++CD16+) monocyte subsets and their expression of co-stimulatory molecules, CD40, CD80, CD86 and inducible costimulatory ligand (ICOSL) in DENV patients and for comparison a healthy control group. In addition, it was the objective to determine if the phenotypic markers of three monocyte subsets as well as their kinetics would help distinguish benign illness of DF from the more severe form of DENV infection including DHF. The results presented herein indicate that while no detectable difference was noted in the frequencies of the non-classical monocytes between the DENV patients and the controls, DENV patients of both DF and DHF possess significantly higher frequency of intermediate monocytes but lower frequency of classical monocytes when compared to the control group. The increased frequency of the intermediate monocytes during the febrile phase gradually increased throughout the course of infection and returned to normal levels during the afebrile phase. The changes in the frequency of the intermediate monocytes were also associated with differences in the kinetics of the frequencies of cells expressing the co-stimulatory molecules CD40, CD80, CD86 and ICOSL when compared to the controls. These findings suggest that a more detailed study of the changes in the expression of genes that are activated including those involved in intracellular signaling pathways within this monocyte subset may help provide clues that can help distinguish DF from DHF/DSS.

## 2. Results

### 2.1. Increased Intermediate Monocytes Frequencies in Dengue Virus (DENV)-Infected Patients at the Febrile Phase and Defervescence Phase

**Figure 1:**
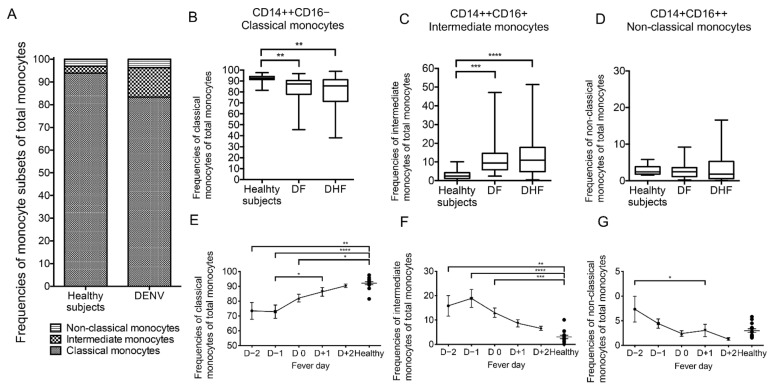
Comparison of the frequencies and their kinetics of monocyte subsets in peripheral blood from dengue virus (DENV)-infected patients and healthy subjects. (**A**) The mean frequencies (%) of classical monocytes, intermediate monocytes and non-classical monocytes were analyzed in blood samples from 68 DENV-infected samples and 14 healthy returned subjects. (**B**–**D**) The frequencies of classical monocytes, intermediate monocytes and non-classical monocytes were compared between the three groups that included healthy subjects (*n* = 14), dengue fever (DF) (*n* = 31) and dengue hemorrhagic fever (DHF) patients (*n* = 37). The box-and-whisker plots indicate the median value (black line in the box) with interquartile range of 25th and 75th percentiles, respectively. (**E**–**G**) The kinetics of the frequencies (%) of classical monocytes, intermediate monocytes and non-classical monocytes on blood samples from DENV-infected patients. DENV samples (*n* = 52) were obtained on different days of fever ranging from febrile phase, day −2 (D −2, *n* = 10) to day −1 (D −1, *n* = 13) to defervescence phase, day 0 (D0, *n* = 15) to day +1 (D +1, *n* = 11) and to afebrile day, day +2 (D +2, *n* = 3). Solid line represents the trend of frequencies of monocyte subsets from DENV patients (mean ± SEM). Dot plot represents the frequencies of monocyte subsets from healthy subjects. Statistical analyses were performed using the Dunn’s post-test after Kruskal–Wallis test. (* *p* < 0.05, ** *p* < 0.01, *** *p* < 0.001 and **** *p* < 0.0001).

The kinetics of changes in the frequencies of classical monocytes (CD14++CD16−), intermediate monocytes (CD14++CD16+) and non-classical monocytes (CD14+CD16++) were first analyzed in the 68 whole blood samples collected from 20 DENV-infected patients (DF and DHF) at various times post-DENV infection regarding different day of fever and the data compared with blood samples from healthy control subjects by standard flow cytometry (Figure 1). The frequency (%) of total monocytes (CD14+ cells) from CD3−/CD19−/CD56− cells of the healthy subjects was median 44.13% (interquartile range [IQR] = 33.75–50.48), while the frequency (%) of total monocytes of DENV-infected patients from the total DENV group was median 52.25% (interquartile range [IQR] = 38.35–62.83). The frequencies of total monocytes were significantly increased in total DENV group (*p* = 0.03) and DHF patients (*p* = 0.02) compared with values obtained on samples from healthy subjects. (Table 1). The mean frequencies (%) of classical monocytes, intermediate monocytes and non-classical monocytes from the peripheral blood from healthy subjects were calculated as 93.85%, 3.11% and 3.04%, respectively, while the mean frequencies (%) of classical monocytes, intermediate monocytes and non-classical monocytes from all 68 peripheral blood samples from the DENV-infected patients were 83.32%, 12.98% and 3.71%, respectively (Figure 1A). All subsequent data represent mean values of each subset on all samples analyzed (from day −2 (D −2) to day +2 (D +2)) from DF and the mean values compared with DHF patients. As seen in Figure 1B and Table 1, the frequencies of classical monocytes were significantly decreased in both DF patients (*p* < 0.01) and DHF patients (*p* < 0.01) compared with values obtained on samples from healthy subjects. We also evaluated the kinetics of the changes in the three monocyte subsets based on samples obtained daily from D −2 (febrile phase) to D +2 (afebrile phase). The kinetics of frequencies of classical monocytes from DENV-infected patients were significantly decreased from day -1 (D −1), febrile phase) to day +1 (D +1), defervescence phase) (*p* = 0.04) (Figure 1E). Moreover, the kinetics of the frequencies of classical monocytes from DENV-infected patients were significantly decreased at D −2 (*p* = 0.003), D −1 (*p* < 0.0001) and D0 (*p* = 0.01) when compared with the mean values obtained from healthy individuals (Figure 1E). In contrast, the frequencies of intermediate monocytes were significantly higher in both the DF patients (*p* < 0.001) and DHF patients (*p* < 0.0001) when compared with values obtained on healthy individuals (Figure 1C and Table 1). As noted, the kinetics of the frequencies of intermediate monocytes from the DENV-infected patients were increased during the febrile phase and then decreased from the defervescence phase to the afebrile phase, but there were no significant differences in the kinetics of changes in the frequencies of intermediate monocytes between days of fever (Figure 1F). Interestingly, the kinetics of frequencies of intermediate monocytes from DENV-infected patients were significantly increased at D −2 (*p* = 0.002), D −1 (*p* < 0.0001) and D0 (*p* = 0.0004) when compared with the mean values obtained from healthy individuals (Figure 1F). While there was no significant difference in the frequencies of non-classical monocytes between healthy individuals and DENV-infected patients (Figure 1D and Table 1), in the kinetic studies the frequency of non-classical monocytes showed a small increase that returned to normal levels during the afebrile phase (Figure 1G). These data indicate that measurements of frequencies and the kinetics by which these changes occur in the three monocyte subsets in dengue patients, while clearly different from values of healthy controls, did not help in distinguishing DF from DHF.

### 2.2. Alteration in the Expression of Co-Stimulatory Molecules on Monocytes during Acute DENV Infection

**Figure 2:**
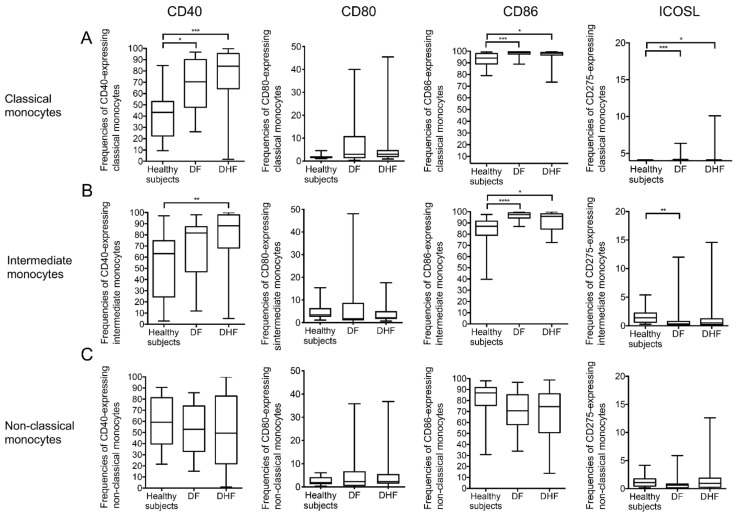
Comparison of the frequencies of co-stimulatory molecules expressed on each monocyte subset during acute DENV infection. The frequencies of CD40, CD80, CD86 and inducible costimulatory ligand (ICOSL) expressed by (**A**) classical monocytes, (**B**) intermediate monocytes and (**C**) non-classical monocytes were compared between healthy subjects (*n* = 14) and DENV-infected patients of both DF (*n* = 31) and DHF patients (*n* = 37). The box-and-whisker plots indicate the median value (black line in the box) with interquartile range of 25th and 75th percentiles, respectively. Statistical analyses were performed using the Dunn’s post-test after the Kruskal–Wallis test for comparison of three groups. (* *p* < 0.05, ** *p* < 0.01, *** *p* < 0.001 and **** *p* < 0.0001).

Since T-cells require two signals for full activation, it was reasoned that the frequencies of each subset expressing the spectrum of co-stimulatory molecules and the relative density of expression of these molecules may provide additional insights on the potential role of these subsets during DENV infection. In addition, we examined these data for their relationship to disease severity. The co-stimulatory molecules expressed by each monocyte subset examined using stand polychromatic flow cytometry included CD40, CD80, CD86, and ICOSL (CD275). We first established the gating strategy for determining the frequency of each subset that expressed the individual co-stimulatory molecule and determined the relative mean fluorescent intensity (MFI) of each molecule. As seen in Figures S1 and S2, there were marked differences in the background noted with isotype controls for each antibody against the co-stimulatory molecules on each of the three subsets. This was taken into account for calculating the frequencies of cells expressing the appropriate co-stimulatory molecule. When the frequencies of CD40 and CD86 expressing monocytes were examined, there appeared to be increased frequencies of both CD40 and CD86 expressing classical and intermediate monocyte subsets but not non-classical monocyte subsets from the DF and DHF patients when compared with values from healthy controls (Figure 2). There did not appear to be any statistically significant difference in the frequencies of CD80 expressing monocyte subsets in samples from DF and DHF when compared to healthy controls. While the frequencies of the three monocyte subsets expressing ICOSL is quite low, some subtle differences were noted. Thus, there was a statistically significant increase in the frequencies of classical and intermediate subset of monocyte expressing ICOSL particularly in samples from DF patients (Figure 2A,B, right panel).

**Figure 3:**
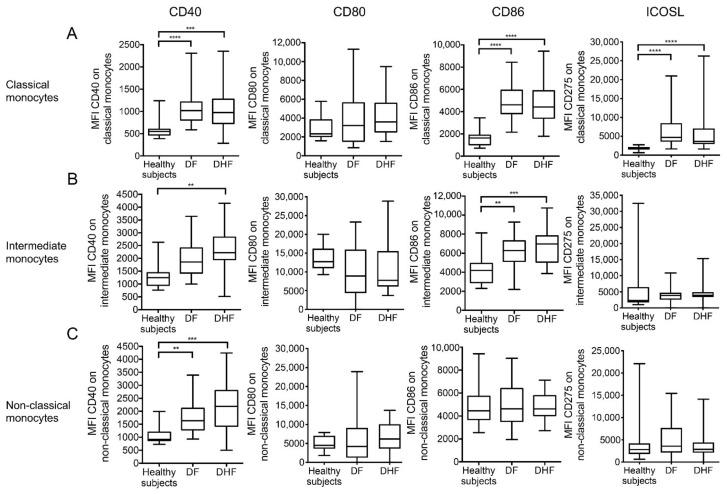
Comparison of the mean fluorescent intensity (MFI) of co-stimulatory molecules expressed on each monocyte subset in DENV-infected patients. Comparative analysis of the MFI of CD40, CD80, CD86, and ICOSL expressed by (**A**) classical monocytes, (**B**) intermediate monocytes, and (**C**) non-classical monocytes in blood samples from healthy subjects (*n* = 14) and DF (*n* = 31) and DHF patients (*n* = 37). The box-and-whisker plots indicate the median value (black line in the box) with interquartile range of 25th and 75th percentiles, respectively. Statistical analyses were performed using the Dunn’s post-test after the Kruskal–Wallis test for comparison of three groups. (** *p* < 0.01, *** *p* < 0.001 and **** *p* < 0.0001).

When the relative MFI levels of co-stimulatory molecule expression by the three monocyte subsets were examined, a number of differences emerged (Figure 3). Thus, there were clearly increased levels of CD40 expressed by all three monocyte subsets from DF and DHF patients as compared with healthy controls (Figure 3, left panel). However, no significant difference was noted in the levels of CD80 expressed by any of the three monocyte subsets. On the other hand, there were significantly higher levels of CD86 expressed by both classical and intermediate subsets of monocytes but not non-classical monocytes when compared with healthy controls (Figure 3, 3rd column). Finally, with regards to ICOSL expression, the only significant difference noted was in the levels of ICOSL expression on classical monocytes from DF and DHF patients when compared with healthy controls (Figure 3A, right panel). Taken together, the data therefore appear to indicate that the major differences in co-stimulatory molecule expression appears to be in the levels of CD40 and CD86 expression by classical and intermediate monocyte subsets from dengue patients when compared with healthy controls but did not distinguish DF from DHF.

### 2.3. Kinetic Changes of Co-Stimulatory Molecules on Monocytes during Acute DENV Infection

**Figure 4:**
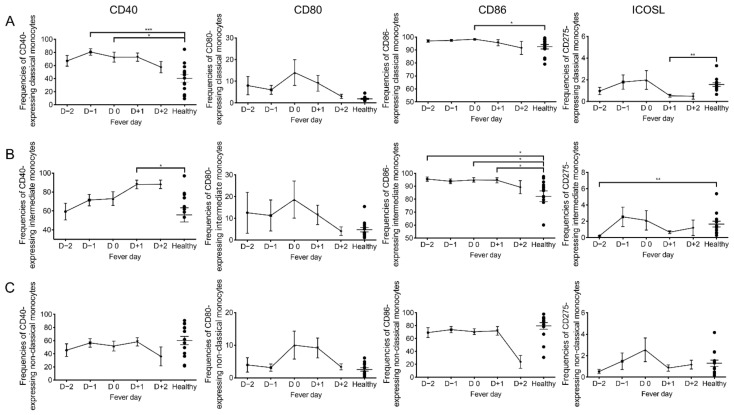
Kinetic changes of the frequencies of co-stimulatory molecules expressed on each monocyte subset during acute DENV infection. The frequencies of CD40, CD80, CD86, and ICOSL expressed by (**A**) classical monocytes, (**B**) intermediate monocytes, and (**C**) non-classical monocytes were determined on DENV samples (*n* = 52) obtained on different days of fever ranging from febrile phase, day −2 (D −2, *n* = 10) to day −1 (D −1, *n* = 13) to defervescence phase, day 0 (D0, *n* = 15) to day +1 (D +1, *n* = 11) and to afebrile day, day +2 (D +2, *n* = 3). Solid line represents the frequencies of CD40, CD80, CD86, and ICOSL on samples from DENV patients (mean ± SEM) compared with healthy subjects (*n* = 14). Statistical analyses were performed using the Dunn’s post-test after Kruskal–Wallis test. (* *p* < 0.05, ** *p* < 0.01 and *** *p* <0.001).

The differences in the frequencies and levels of the CD40, CD86, and ICOSL co-stimulatory molecules expression on monocyte subsets during acute DENV infection prompted us to examine in more detail the kinetics by which these changes occur on DENV samples collected from D −2 to D +2 and compare these values with mean values from healthy controls (Figure 4). Since there were no detectable differences in the frequencies and the densities of co-stimulatory molecules expressed by the three monocyte subsets from DF as compared with DHF, the data from these two groups of patients were pooled and described as derived from dengue infected patients. While there were increases in the frequencies on both classical and intermediate monocyte subsets expressing CD40 from D −1 to D +1, these cell lineages did not return to baseline at D +2 (Figure 4A,B), there was a small but sustainable decrease in the frequencies of non-classical monocyte subset as a function of time post infection (Figure 4C). Although there were no significant differences in the kinetics of changes in the frequencies of CD40 on these three monocyte subsets between days of fever (Figure 4A–C), there were significantly increased on classical monocytes at D −1 (*p* < 0.001) and D0 (*p* = 0.01) and intermediate monocytes at D +1 (*p* = 0.009) when compared with the mean values obtained on healthy individuals (Figure 4A,B).

When the frequencies of CD80 expressing monocyte subsets was examined, it was of interest to note that there was a trend (but statistically insignificant) for increases in all three monocyte subsets that appear to peak on D0 that returned back to baseline by D +2. While there was no significant change in the frequencies of CD86 of the three monocyte subsets of DENV patients based on days of fever, there were significantly increased of CD86 expression by the classical and intermediate monocytes when compared with the mean values obtained from healthy individuals (Figure 4). There was a slight decrease of CD86 expression by the non-classical monocytes. However, it should be kept in mind that the frequencies of non-classical monocytes are extremely low and thus the significance of this observation thus remains questionable. Finally, when the frequencies of ICOSL expressing monocyte subsets was examined as a function of time post-infection, there was no significant difference noted for all three subsets from D −2 to D +2 (Figure 4, right panel).

**Figure 5:**
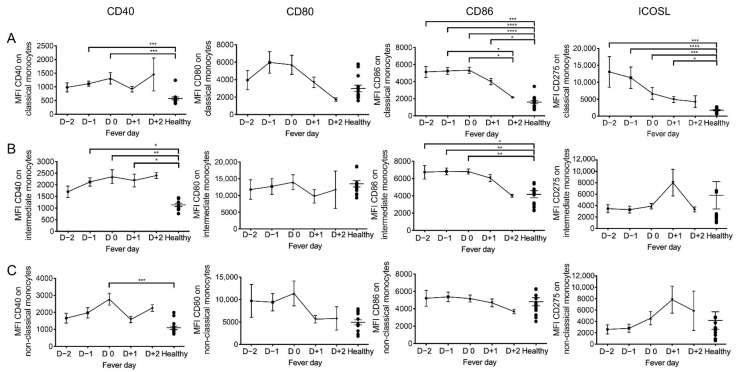
Kinetic changes of the MFI of co-stimulatory molecules expressed by each monocyte subset during acute DENV infection. The MFI of CD40, CD80, CD86, and ICOSL expressed by (**A**) classical monocytes, (**B**) intermediate monocytes, and (**C**) non-classical monocytes were determined on DENV samples (*n* = 52) obtained on different days of fever ranging from febrile phase, day –2 (D –2, *n*=10) to day –1 (D –1, *n* = 13) to defervescence phase, day 0 (D0, *n* = 15) to day +1 (D +1, *n* = 11) and to afebrile day, day +2 (D +2, *n* = 3). Solid line represents the MFI of CD40, CD80, CD86, and ICOSL on samples from DENV patients (mean ± SEM) compared with healthy subjects (*n* = 14). Statistical analyses were performed using the Dunn’s post-test after Kruskal–Wallis test. (* *p* < 0.05, ** *p* < 0.01, *** *p* <0.001 and **** *p* <0.0001).

Next, we examined the kinetics by which changes occurred in the relative density of co-stimulatory molecule expression (Figure 5). There appeared to be a tendency for an increase in the mean fluorescence intensity (MFI) of CD40 expression on all three monocyte subsets as a function of time post infection from D −2 to D +2 (Figure 5, left panel). The MFI of CD80 expression on classical monocytes showed a transient increase during D −1 and D0 that returned back to baseline on D +1 and D +2. While there was no statistical difference noted in the MFI expressed by intermediate monocytes, the MFI of CD80 expression by non-classical monocytes showed a sharp decrease by D +1 that remained low on D +2. The MFI of CD86 on both classical monocytes and intermediate monocytes were markedly increased on D −2, D −1 and D0, but they gradually decreased on D +1 and returned to baseline on D +2 (Figure 5). There was no significant changes in the kinetics of MFI of CD86 expression on non-classical monocytes. The levels of ICOSL expressed by the three monocyte subsets showed a markedly different pattern. Thus, while there was a marked increase in the MFI of ICOSL expression by classical monocytes on D −2, these levels gradually decreased to almost baseline levels by D +2. On the other hand, there was a marked decrease (compared with healthy controls) of ICOSL expression on intermediate and non-classical monocytes but they appeared to return to normal levels by D +1 and D +2.

Attempts to correlate the kinetics by which changes occur in both the frequency and relative density of co-stimulatory marker expression suggest that while both the frequencies and MFI of CD40 expressing classical and intermediate monocytes increase throughout the sampling times of dengue infection (D −2 to D +2), there is no change in the frequency but an increase in the MFI expressed by non-classical monocytes from D −2 to D +2 (Figure 4 and Figure 5). With regards to data on CD80 expressing monocytes, these data showed the most striking difference with increases in both the frequencies and MFI of CD80 expression by classical monocytes. There was considerable variability in the data on the expression of CD80 by intermediate and non-classical monocytes to derive any meaningful conclusion. Of interest was the finding that while there was no meaningful increase in the frequencies of the three monocyte subsets expressing CD86, there was clearly an increase in both the frequencies and MFI of CD86 expression on both classical and intermediate monocytes but not non-classical subsets. Finally, while the frequencies of ICOSL expression by the three monocyte subsets does not change appreciably, there is clearly a marked decrease in the MFI of ICOSL expression by intermediate and non-classical monocytes at least during the febrile phase of dengue infection.

## 3. Discussion

Monocytes are important innate immune cells not only as progenitors for tissue macrophages but also for their contribution to the process of antigen processing and presentation. They express co-stimulatory molecules, produce cytokines, and activate subsequent adaptive immunity. They are potent phagocytic cells responsible for killing of invading microorganisms including DENV. These blood mononuclear phagocytes/tissue macrophages are distinguished from other white blood cells by their expression of CD14 and have also long been recognized as the prime target and a major reservoir for DENV infection and replication [14,15,16,17,20]. This cell lineage is known to play an important role in pathogenesis since they are known to produce several inflammatory cytokines including those that lead to endothelial dysfunction and vascular leakage (a major pathologic sequelae in select cases of DENV infection. Moreover, they also express Fc-γ receptors of CD16 that potentially contribute to ADE during secondary DENV infections [5,6,21], although this has been a subject of considerable controversy. In the present study, we characterized differences in the frequencies of monocyte subsets using phenotypic markers and attempted to further characterize these subsets by the expression of co-stimulatory molecules with the hopes that we could identify changes that can distinguish DF from DHF/DSS. The studies also included longitudinal studies in DF and DHF patients in efforts to identify specific time periods that could be potentially utilized for modulating DENV pathogenesis.

Using panels of mAbs that facilitate the identification of subsets of monocytes and their co-stimulatory molecules, we found that while DENV did not change the frequency of the non-classical monocytes, the infection led to a significant increase in the frequency of intermediate monocytes but a concomitant decrease in the frequency of classical monocytes consistent with other previous reports [22,23]. Our findings are also in partial agreement with other studies that have shown a decrease in the frequencies of both classical and non-classical monocytes that is compensated for by an increase in the frequency of intermediate monocytes in patients with DENV infection [19,24]. Some of the discrepancies could be explained by the different grades of DENV severity, day of blood collection and the difference in the flow cytometric gating strategies used, as our study characterized monocytes subsets based on cells that expressed CD45+CD3-CD19-CD14+CD16+/-CD56-. Since classical monocytes account for about 80–95% of normal peripheral blood monocytes, a decrease in their frequency could be secondary due to their preferential infection by DENV and that the infection subsequently activates and alters the homeostasis of the short life span of these cells [25]. It is important to keep in mind that changes in the frequencies of monocyte subsets in the blood could also be due to differences in the trafficking of the cells from tissues affected by DENV infection [26]. Changes in chemokines and chemokine receptors involved in the trafficking of NK cells, for example, has been shown to occur during acute DENV infection [27]. It has been shown that DENV-infected human monocytes in vitro triggers cell death related to the production of apoptotic-type of cytokines even as early as 6 h post infection [28,29]. While our study showed different kinetics of changes in monocyte subsets after the first evidence of fever, our study showed that while the frequency of classical monocytes was low during the febrile phase (D −2 to D −1), this frequency gradually increased during the defervescence and afebrile phase of D +1 or D +2. This was in contrast with the kinetic changes of both intermediate and non-classical monocytes in which both monocyte subsets showed a continuous decrease in the frequency from febrile phase to the afebrile phase. Therefore, our results suggest that during the acute phase of infection, the classical monocytes which are known to both control and disseminate the virus, upon encounter and/or following infection by DENV, become activated and produce apoptosis-inducing cytokines leading to the death of these affected classical monocytes. It would be interesting if the depletion of these classical monocytes is related to an increase in systemic DENV titers as shown in a murine model of DENV infection [30]. For intermediate monocytes, an increase in the frequency during the early phase of symptomatic disease suggests a pivotal role of these cells in mediating humoral immunity to acute DENV infection [19], and is supported by results of our previous study that most of the antibody synthesizing cells are activated and become plasmablasts that secrete antibodies during the early phase of infection but are then specifically eliminated during the late stages of infection [31]. DENV-infected patients classified as those with DF and DHF showed a decrease in the frequency of classical monocytes concomitantly with an increased levels of co-stimulatory molecule expression, especially CD40, CD86 and ICOSL but not CD80 molecules. This result may suggest that the decreased classical monocyte population of DENV patients may have an increased capacity of presenting antigens to adaptive immune cells through the increased expression of these co-stimulatory signals. It is possible that upregulation of these co-stimulatory markers on affected monocytes may be a consequence of increased exposure to both DENV antigens and inflammatory cytokines such as TNF-α and IFN-α [32,33]. In addition, both CD80 and CD86 are molecules with similar structure and function and expressed by antigen-presenting cells such as monocytes/macrophages and are known to play a major role in the outcome of T-cell activation [34]. Thus, the distinct patterns of expression and their role in the homeostasis of monocytes are different. While the CD86 molecule has a higher affinity for its receptor CD28 and is involved in productive T-cell activation, IL-2 secretion and cell survival through enhancing Bcl-2 expression [35], CD80 has higher relative affinity for CD152 associated with an opposing activity by inhibiting T-cell activation, favoring apoptosis and inducing cell anergy or tolerance [36]. These data appear to suggest that high levels of CD40, CD86 and ICOSL expression by the majority of monocytes leads to activation of T-cells and the induction of inflammatory cytokines that have been characterized as “cytokine storms” and thought to be a hallmark of infection with a heterologous dengue virus in previously DENV exposed patients. Further studies on the function and interaction with the adaptive T- and B-cells of these three monocyte subsets could provide a better understanding of the pathogenesis of DENV infection.

One of the major objectives of our studies was to attempt to identify changes in the frequencies of subsets and/or expression of co-stimulatory molecules by the subsets that could help distinguish DF from DHF/DSS. Unfortunately, none of the data sets we obtained showed a statistical difference, although some trends in the changes were noted. There have been exhaustive attempts to identify markers that can distinguish DF from DHF/DSS because of its important clinical implications. These previous attempts including the present study seem to suggest that perhaps ultra-sensitive single cell multiomic profiling including those involved in immunometabolism [37] of each of these monocyte subsets may provide some clues that are not readily apparent using the techniques that have been so far utilized. These thoughts are supported by the studies that have documented additional monocyte subsets based on gene and protein expression patterns [38,39,40] and is consistent with the fact that increased severity of disease has been shown to be associated with increased frequencies of non-classical and intermediate monocyte subsets [41,42] and confirmed herein. If, indeed, unique markers/intra-cellular signaling pathways and/or proteins are identified that distinguish DF from DHF/DSS, particularly the kinetics by which these occur, it is possible that attempts to mitigate may require re-programming of the specific monocyte subset as a potential therapeutic target.

## 4. Materials and Methods

### 4.1. Study Sites and Study Population

Pediatric patients with DENV infection were enrolled in this study upon admission to the Ramathibodi and Siriraj Hospitals, Bangkok, Thailand. This study was approved by the Siriraj Institutional Review Board, Faculty of Medicine Siriraj Hospital, Mahidol University (Si 522/2015). The permission and written informed consent were obtained from parents of participating children prior to initiation of the study. Blood samples were collected from the 20 DENV-infected patients on the day of study admission and daily thereafter until hospital discharge. These collections resulted in 68 whole blood samples from DENV patients that were collected at different days of fever ranging from day −2 (D −2) to day +2 (D +2). Day 0 (D0) was defined as the day of defervescence, when the temperature dropped below 37.5 °C to 38 °C without further temperature elevation. D −2 to D −1 samples were considered as the febrile phase. D0 to D +1 samples were assigned as the defervescence phase, and D +2 sample was considered as the afebrile phase. Blood samples from 14 age-matched healthy volunteer subjects (eight males and six females) were enrolled in this study as controls.

### 4.2. Dengue Diagnostics and Classification

Patients were diagnosed by physicians using standard clinical criteria and routine laboratory tests. The laboratory tests included complete blood count, urinalysis, and blood chemistry. DENV infection was confirmed by serological diagnosis using the PlateliaTM Dengue non-structural protein 1 (NS1) Ag microplate EIA (Hercules, CA, USA). DENV serotype were determined by reverse transcription-polymerase chain reaction (RT-PCR). Viral RNA was extracted from the DENV-infected plasma sample by using a QIAamp viral RNA extraction kit (Qiagen, Germany) according to the manufacturer’s protocol. The four DENV serotypes was performed using a multiplex nested RT-PCR. Briefly, the extracted RNA was converted to cDNA by reverse transcriptase. The PCR product was subjected to a multiplex nested PCR using the appropriate primers that target Env region (Table S1). Twenty DENV-infected patients were classified as DF (*n* = 11) and DHF (*n* = 9) according to the WHO 1997 guidelines (Table 2).

### 4.3. Monoclonal Antibodies

Immunofluorescence staining of monocyte subsets and their co-stimulatory molecules were performed by using fluorochrome-conjugated monoclonal antibodies (mAbs) consisted of anti-CD3-fluorescein isothiocyanate (FITC) (clone SK7, T-cell marker); anti-CD19-PE-Texas Red (clone SJ25-C1); anti-CD86-PE/Dazzle594 (clone IT2.2); anti-CD45-peridinin chlorophyll protein (PerCP) (clone 2D1); anti-CD275(ICOSL)-allophycocyanine (APC) (clone 2D3); anti-CD3-allophycocyanin/cyanine 7 (APC-Cy7) (clone SK7); anti-CD19-APC-Cy7 (clone HIB19); anti-CD56-APC-Cy7 (clone HCD56); anti-CD40-Pacific Blue (clone 5C3); anti-CD16-BV510 (clone 3G8); anti-CD14-BV570 (clone M5E2) and anti-CD80-BV605 (clone 2D10). Human IgG isotype control antibodies conjugated with APC, Pacific Blue, PE/Dazzle594 and BV605 were used for each specific co-stimulatory molecules. Fluorochrome-conjugated mAbs were purchased from Biolegend (San Diego, CA, USA), except anti-CD3-FITC and anti-CD45-PerCP that were purchased from BD Biosciences (San Jose, CA, USA) and anti-CD19-PE-Texas Red from Southern Biotech (Birmingham, AL, USA).

### 4.4. Extracellular Staining

Fresh whole blood samples were aliquoted and incubated with fluorochrome-conjugated mAbs (Table S2) for 15 min at room temperature. After incubation, red blood cells were lysed with 1X FACSlysing solution (BD Biosciences) for 5 min. Cells were washed with phosphate buffered saline (PBS) twice. The stained samples were re-suspended in PBS and analyzed by using multi-color flow cytometric analysis.

### 4.5. Flow Cytometric Analysis of Peripheral Blood

**Figure 6:**
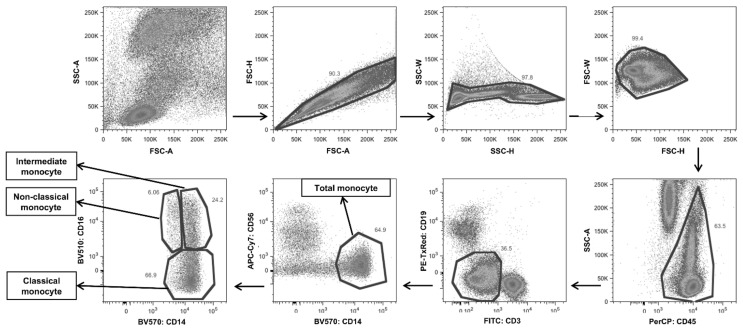
Representative flow cytometric gating strategy of monocytes from peripheral blood of DENV-infected patient. After exclusion of doublets, the gated population of CD45+ cells that expressed CD3−/CD19−/CD56−/CD14+ cells were identified as monocytes. The three monocyte subsets were identified based on their levels of expression of CD14 and CD16. Monocyte subsets were identified as classical monocytes (CD14++CD16−), intermediate monocytes (CD14++CD16+) and non-classical monocytes (CD14+CD16++). The gating strategy of CD45+ cells was adapted from Lertjuthaporn et al. [43].

Samples were acquired on a LSRFortessa flow cytometer (BD Bioscience) and analyzed with FlowJo software (Tree Star, Ashland, OR, USA). Flow cytometry gating strategy for CD45+ cells in DENV-infected patients (Figure 6) and healthy individual (Figure S3) was performed according to our previous publication [43]. In which white blood cells were initially analyzed for their FSC-A/FSC-H, SSC-H/SSC-W, and FSC-H/FSC-W to eliminate doublets. The lymphocytes and monocytes were then gated based on their SSC-A/bright CD45+. Total monocytes were defined as CD14+. Monocyte subsets were further divided into three subsets that included classical monocytes (CD14++CD16−), intermediate monocytes (CD14++CD16+) and non-classical monocytes (CD14+CD16++). The mAbs against CD40, CD80, CD86, and ICOSL were used to define co-stimulatory molecules expressed by these three monocyte subsets and the use of isotype controls to define background fluorescence. Flow cytometric data were expressed as frequency (%) and mean fluorescence intensity (MFI) of cells expressing each antigen.

### 4.6. Statistical Analysis

Statistical analyses were performed using GraphPad Prism 8.4.0 software. Kruskal–Wallis tests with Dunn’s multiple comparison post-tests were used to compare data on more than two groups. *p* values < 0.05 were considered statistically significant.

## 5. Conclusions

In conclusion, we have provided evidence that there are different patterns of the three monocyte subsets during acute DENV infection. Although we could not observe any association between the changes of these subsets of monocytes and the severity of DENV disease, we found that while there is no change in the frequency of non-classical monocytes, there are changes in the other two monocyte subsets in DENV patients when compared to the control subjects. These changes consist of a significant decrease in classical monocytes and an increase in intermediate monocytes. Interestingly, we found that the different patterns of these two major monocyte subsets that occur during the early phase of infection gradually return to normal and are accompanied by a significant up-regulation of some co-stimulatory molecule CD40, CD86 and ICOSL during the febrile and defervescence phase. An increase in these co-stimulatory molecules on the majority of peripheral blood monocytes indicates that these monocytes from DENV-infected patients may have increased antigen-presenting function as shown by increased co-stimulatory signals. Taken together, these data may provide a useful understanding of monocyte subsets distribution and their longitudinal fate based on their frequencies and co-stimulatory markers during the course of DENV infection.

## Figures and Tables

**Table 1:** Comparison of the frequencies of total monocyte and monocyte subsets from DENV-infected patients and healthy subjects.

Monocyte	Healthy Subjects	DENV-Infected Patients	
		DF	DHF
Total monocytes (%)	44.13 (33.75–50.48)	53.90 (40.80–68.90)	60.60 (46.70–66.05)
Classical monocytes (%)	92.55 (91.30–94.13)	87.30 (77.90–90.50)	85.60 (71.35–91.15)
Intermediate monocytes (%)	2.52 (1.26–4.25)	9.46 (5.82–14.60)	11.00 (4.81–17.75)
Non-classical monocytes (%)	2.43 (1.79–3.83)	2.45 (1.13–3.61)	1.80 (0.58–16.60)

The data are provided as median (interquartile range).

**Table 2:** Demographic characterization of the study population.

Subjects	DENV Patients		Healthy Individuals
	DF	DHF (Grade I, II, III)	
Number	11	9 (4, 3, 2)	14
Gender			
Male	5	5	8
Female	6	4	6
Mean of Age (range)	13 (10–18)	12 (7–14)	10 (3–13)
DENV serotypes			
DENV-1 (*n* = 3)	1	2	−
DENV-2 (*n* = 3)	2	1	−
DENV-3 (*n* = 5)	2	3	−
DENV-4 (*n* = 9)	6	3	−

DENV, dengue virus; DF, dengue fever; DHF, dengue hemorrhagic fever; grade, levels of severity followed 1997 WHO criteria.

## Data Availability

The data presented in this study are available on request from the corresponding author.

## Supplementary Materials

The following are available online at https://www.mdpi.com/article/10.3390/pathogens10111458/s1, Supplementary Figure S1: Gating strategy of co-stimulatory molecules expressed on each monocyte subset in healthy subjects, Supplementary Figure S2: Gating strategy of co-stimulatory molecules expressed on each monocyte subset during acute DENV infection, Supplementary Figure S3: Representative flow cytometric gating strategy of monocytes from peripheral blood of healthy subject, Supplementary Table S1: Primer sequences for amplification of dengue viral RNA, Supplementary Table S2: Surface staining panels for the phenotypic characterization of monocytes and their co-stimulatory molecules.
