# Supplementary material for: Kinetic Changes of Peripheral Blood Monocyte Subsets and Expression of Co-Stimulatory Molecules during Acute Dengue Virus Infection
Source: Pathogens. 2021 Nov 10;10(11):1458. doi: 10.3390/pathogens10111458 (PMC8625762; doi:10.3390/pathogens10111458)
Supplement: Supplementary file 1 [file pathogens-10-01458-s001.zip › pathogens-1429591-supplementary.pdf]

pathogens

MDPI

Supplementary Materials

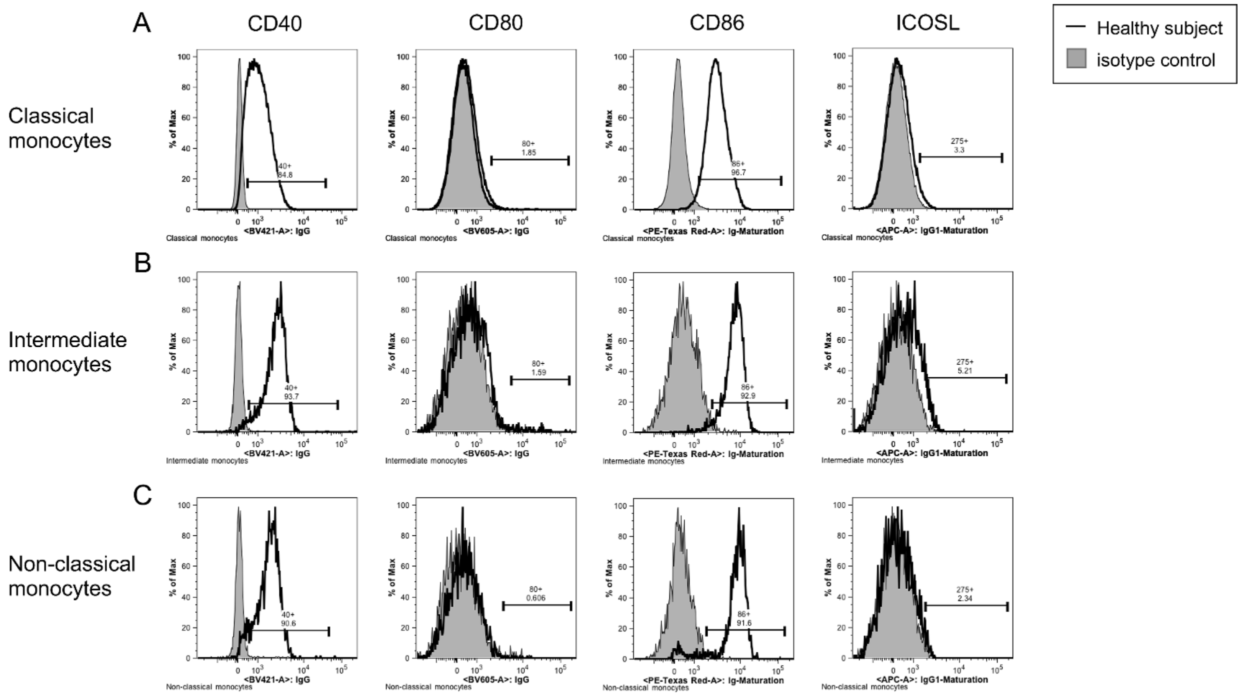

Figure S1. Gating strategy of co-stimulatory molecules expressed on each monocyte subset in healthy subjects. Representative histogram plots display the co-stimulatory molecules CD40, CD80, CD86 and ICOSL expressed by (**A**) classical monocytes, (**B**) intermediate monocytes and (**C**) non-classical monocytes on blood samples from healthy subjects (solid black line) and showing their isotype control (shaded grey).

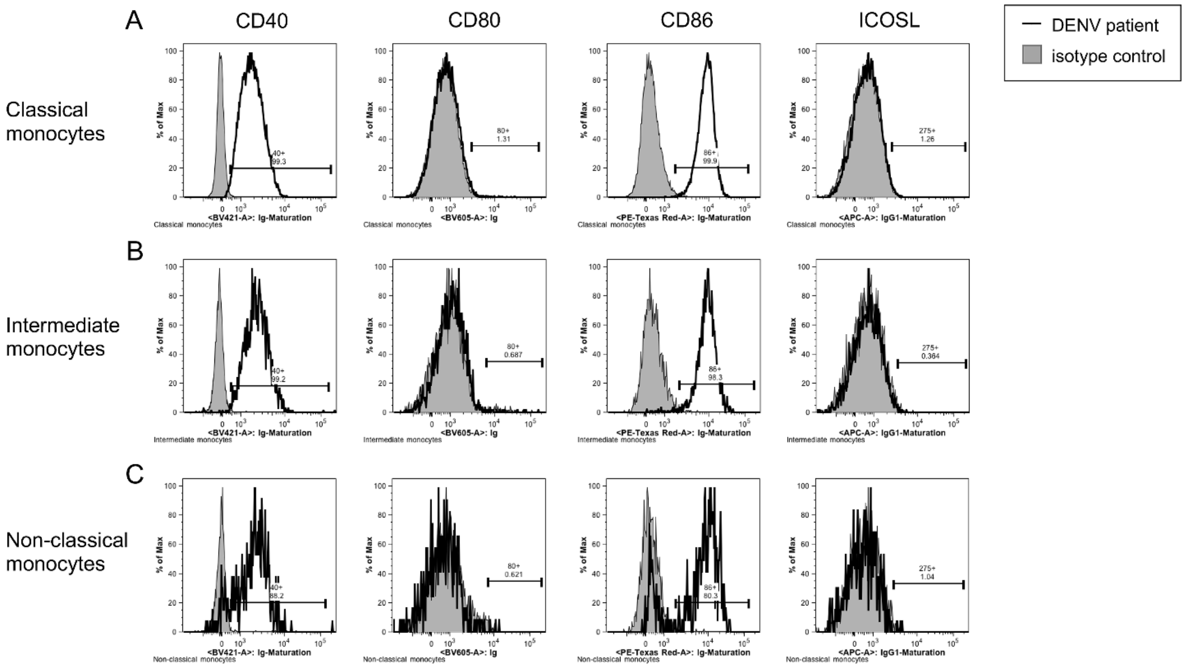

Figure S2. Gating strategy of co-stimulatory molecules expressed on each monocyte subset during acute DENV infection. Representative histogram plots display the co-stimulatory molecules CD40, CD80, CD86 and ICOSL expressed by (**A**) classical monocytes, (**B**) intermediate monocytes and (**C**) non-classical monocytes on blood samples from a representative DENV-infected patient (solid black line) and showing their isotype control (shaded grey).

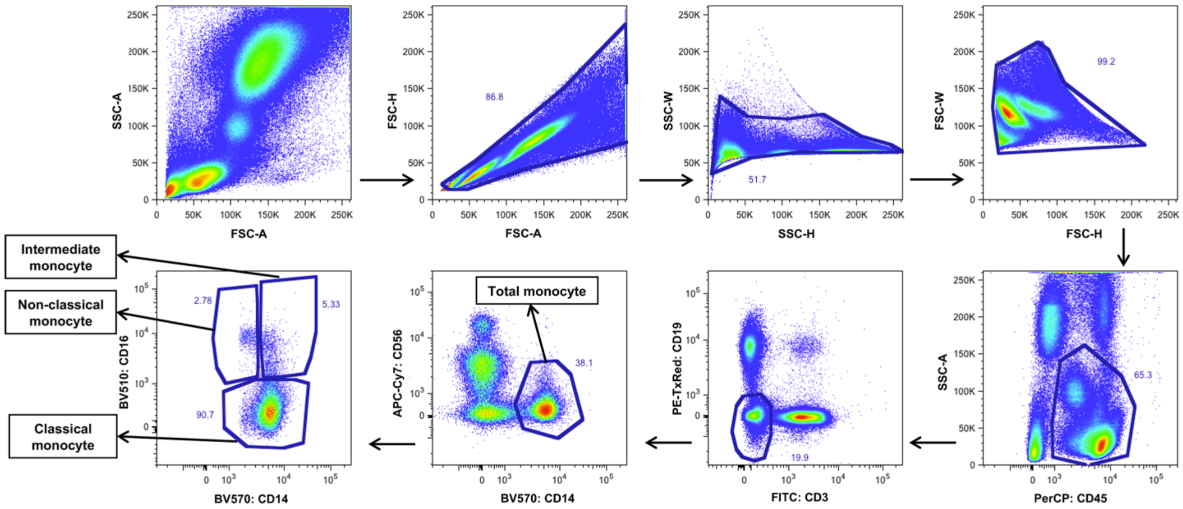

Figure S3. Representative flow cytometric gating strategy of monocytes from peripheral blood of healthy subject. After exclusion of doublets, monocytes were identified on the gated population of CD45^{+} cells. The CD45+/CD3−/CD19−/CD56−/CD14+ cells were further analyzed for monocyte subsets based on their levels of expression of CD14 and CD16. Monocyte subsets were identified as classical monocytes (CD14++CD16−), intermediate monocytes (CD14++CD16+) and non-classical monocytes (CD14+CD16++). The gating strategy of CD45+ cells was adapted from Lertjuthaporn et al. [43].

Table S1. Primer sequences for amplification of dengue viral RNA1

Primer	Sequence	Product Size (bp)
E region		
DEUL	5′-TGGCTGGTGCACAGACAATGGTT-3′	641
DEUR	5′-GCTGTGTCACCCAGAATGGCCAT-3′	
Type-specific		
D1L	5′-GGGGCTTCAACATCCCAAGAG-3′	504
D1R	5′-GCTTAGTTTCAAAGCTTTTTCAC-3′	
D2L	5′-ATCCAGATGTCATCAGGAAAC-3′	346
D2R	5′-CCGGCTCTACTCCTATGATG-3′	
D3L	5′-CAATGTGCTTGAATACCTTTGT-3′	196
D3R	5′-GGACAGGCTCCTCCTTCTTG-3′	
D4L	5′-GGACAACAGTGGTGAAAGTCA-3′	143
D4R	5′-GGTTACACTGTTGGTATTCTCA-3′	

Table S2. Surface staining panels for the phenotypic characterization of monocytes and their co-stimulatory molecules1

Panel for monocytes	FITC	PE-TxRd	PerCP	APC	APC-Cy7	Pacific Blue	BV510	BV570	BV605
**Frequency**	CD3	CD19	CD45	–	CD56	–	CD16	CD14	–
**Co-stimulatory**	–	CD86*	CD45	CD275	CD3+19+56	CD40	CD16	CD14	CD80

PE-TxRd, PE-Texas Red; *CD86-PE/Dazzle594.
